# CD8^+^*T* cells from patients with cirrhosis display a phenotype that may contribute to cirrhosis-associated immune dysfunction

**DOI:** 10.1016/j.ebiom.2019.10.011

**Published:** 2019-10-31

**Authors:** Fanny Lebossé, Cathrin Gudd, Enes Tunc, Arjuna Singanayagam, Rooshi Nathwani, Evangelos Triantafyllou, Oltin Pop, Naveenta Kumar, Sujit Mukherjee, Tie Zheng Hou, Alberto Quaglia, Fabien Zoulim, Julia Wendon, Ameet Dhar, Mark Thursz, Charalambos G. Antoniades, Wafa Khamri

**Affiliations:** a Division of Integrative Systems Medicine and Digestive Diseases, Department of Surgery and Cancer, St. Mary's Campus Imperial College London, London, United Kingdom; b Institute of Liver Studies, King's College Hospital, King's College London, United Kingdom; c INSERM U1052- Cancer Research Centre of Lyon (CRCL), 69003 Lyon, France; d Institute of Immunity and transplantation, University College London, United Kingdom

**Keywords:** Cirrhosis-associated immune dysfunction, CD8^+^*T* cells, Chronic liver disease, ACLF, acute-on-chronic-liver disease, AD, acute decompensation, ALD, alcohol-related liver disease, AAH, acute alcoholic hepatitis, CAID, cirrhosis-associated immune dysfunction, CLD, Chronic liver disease, ELD, End-stage-liver diseases, HV, Healthy volunteers, NASH, non-alcoholic steatohepatitis, PMNs, polymorphonuclear neutrophils

## Abstract

**Background:**

Cirrhosis-associated immune dysfunction (CAID) contributes to high sepsis risk in patients with chronic liver disease. Various innate and; to a lesser extent; adaptive immune dysfunctions have been described as contributors to CAID leading to immune-paresis and impaired anti-microbial response in cirrhosis. In this study, we examined the phenotype of CD8^+^*T* cells in chronic liver disease with the aim to evaluate changes that might contribute to impaired immune responses.

**Methods:**

Sixty patients with cirrhosis were prospectively recruited for this study. CD8^+^*T* cells from peripheral blood, ascites and liver explants were characterized using flow cytometry and immunohistochemistry, respectively. The transcriptional signature of flow-sorted HLA-DR^+^CD8^+^*T* cells was performed using Nanostring™ technology. HLA-DR^+^CD8^+^*T* cells interactions with PBMCs and myeloid cells were tested *in vitro*.

**Findings:**

Peripheral CD8^+^*T* cells from cirrhotic patients displayed an altered phenotype characterized by high HLA-DR and TIM-3 surface expression associated with concomitant infections and disease severity, respectively. Paired peritoneal CD8^+^*T* cells expressed more pronounced levels of HLA-DR and PD-1 compared to peripheral CD8^+^*T* cells. HLA-DR^+^CD8^+^*T* cells were enriched in cirrhotic livers compared to controls. TIM-3, CTLA-4 and PD-1 levels were highly expressed on HLA-DR^+^CD8^+^*T* cells and co-expression of HLA-DR and PD1 was higher in patients with poor disease outcomes. Genes involved in cytokines production and intracellular signalling pathways were strongly down-regulated in HLA-DR^+^CD8^+^*T* cells. In comparison to their HLA-DR^−^ counterparts, HLA-DR^+^CD8^+^*T* cells promoted less proliferation of PBMCs and induced phenotypic and functional dysfunctions in monocytes and neutrophils *in vitro*.

**Interpretation:**

In patients with cirrhosis, CD8^+^*T* cells display a phenotypic, functional and transcriptional profile which may contribute to CAID.

**Fund:**

This work was supported by Medical Research Council, the Rosetrees Charitable Trust, Robert Tournut 2016 grant (Sociéte Nationale Française de GastroEntérologie), Gilead® sciences, and NIHR Imperial Biomedical Research Centre.

**Research in context**

**Evidence before this study**

Infections represent a major issue in the management of patients with end-stage liver diseases (ELD). Cirrhosis-associated immune dysfunction (CAID) contributes to high sepsis risk and poor outcomes in patients with cirrhosis. We and others have shown the importance of innate immune dysfunction in the pathogenesis of infection susceptibility in acute and chronic liver failure. However, the impact of adaptive immune defects has not been fully explored. Here, we evaluate and characterize CD8^+^ *T* cells in patients with cirrhosis.

**Added value of this study**

We show that in patients with cirrhosis, total CD8^+^ *T* cells express an activated dysfunctional profile characterized by an expansion of an immunosuppressive HLA-DR^+^CD8^+^ *T* cell subset in peripheral, peritoneal and intrahepatic compartments. HLA-DR expression by CD8^+^ *T* cells was higher in patients who developed infection compared to the ones who did not. Co-expression of PD-1 and HLA-DR was associated with poor outcomes. We reveal that HLA-DR^+^CD8^+^ *T* cells exhibit a down-regulation of genes involved in pro-inflammatory cytokines production and intracellular signalling pathways with the capacity to promote low proliferative responses in autologous peripheral blood mononuclear cells (PBMCs) and to induce dysfunctions in myeloid cells.

**Implications of all the available evidence**

We reveal that in patients with cirrhosis, CD8^+^ *T* cells display a phenotypic, functional and transcriptional profile that may impact susceptibility to infection and disease outcome. Further studies are needed to determine circulating soluble factors involved in the expansion of HLA-DR^+^CD8^+^ *T* cell and to identify targets to counteract adaptive immune defects in cirrhosis.

Alt-text: Unlabelled box

## 1 Introduction

Infections represent a turning point in the natural progression course of cirrhosis and are the main precipitant event for liver insufficiency associated with multi-organ failure, a condition referred to as “Acute-on-chronic liver failure” (ACLF) [1], [2], [3], [4]. Increased susceptibility to infection and the severe prognosis of septic episodes have been associated with cirrhosis-associated immune dysfunction (CAID); a dynamic pattern of immune responses shifting from a predominantly pro-inflammatory to an anti-inflammatory compensatory response [5]. Innate immune dysfunctions in CAID have been well described. In patients presenting alcohol-related liver diseases (ALD), profound impaired oxidative burst and bactericidal functions of polymorphonuclear neutrophils (PMNs) and monocytes were observed [6], [7], [8], [9], [10]. In patients with acute liver failure (ALF) and ACLF, pro-inflammatory conditions could drive an *in vivo* anti-inflammatory monocyte phenotype which was associated with a defective anti-bacterial response *in vitro* [11], [12], [13]. We recently identified that monocytes from ALF and ACLF patients, and to a lesser extent from patients with cirrhosis without organ failure, exhibit an immune regulatory HLA-DR^High^MER-TK^+^ phenotype [11], [12]. Additionally, we reported an expansion of myeloid-derived suppressors CD14^+^CD15^−^HLA-DR^−^ cells in patients with ACLF [13].

Although partially explored, adaptive immune defects are important drivers of immune-paresis and susceptibility to infection in liver diseases. We and others have provided evidence that impaired antimicrobial responses in ALF and alcoholic acute hepatitis (AAH) were mediated by an altered phenotype of CD4^+^ and CD8^+^ *T* lymphocytes characterized by elevated levels of immune checkpoints PD-1, TIM-3 and CTLA-4 [10], [14].

CD8^+^ *T* cells can display a dysfunctional profile induced by chronic antigen stimulation in the context of chronic viral infections or tumours [15]. Recently, a new subset of regulatory CD8^+^ *T* cells with suppressive properties *in vitro* has been discovered in peripheral blood of healthy volunteers (HV) and umbilical blood of new-borns, sharing activated and exhausted CD8^+^ *T* cells characteristics, such as HLA-DR, CTLA-4 and PD-1 surface expression [16], [17].

This study provides a detailed phenotypic, functional and transcriptional characterization of CD8^+^ *T* cells in cirrhotic patients. We reveal new insights on the impact of HLA-DR^+^CD8^+^ *T* cells on CAID.

## 2 Materials and methods

### 2.1 Patients

A total of 60 patients with end stage liver diseases (ELD) were prospectively recruited from February 2016 to October 2017 (Table 1). Twenty five patients with compensated cirrhosis (chronic liver disease (CLD)) were recruited to the study from outpatient hepatology clinics, Imperial College NHS Healthcare Trust. Cirrhotic patients with acute decompensation (AD; *n* = 35) were enrolled within 48 h following admission. Twenty seven healthy volunteers (HV) served as healthy controls. Patients with cirrhosis were included based on liver biopsy, clinical presentation suggestive of cirrhosis and/or on radiological assessment. Clinical data were collected for 1 year after enrolment, with 5 patients being lost to follow-up. Underlying liver diseases and exclusion criteria are described in Supporting Methods.

**TABLE 1:** Clinical characteristics^1^.

	ALL Patients (*n* = 60)	CLD (*n* = 25)	AD (*n* = 35)
Age * (years)^2^	54.6 (48.3 – 63.0)	56.3 (52.7 – 71.3)	52.4 (44.6 – 58)
Sex (Male/ total (n))	36 / 60	15 / 25	21 / 35
ALD (ALD / total (n))	47 / 60	18 / 25	29 / 35
Sepsis (sepsis / total (n))	14 / 60	0 / 25	14 / 35
Cause of AD AAH / sepsis / other / undetermined (n)	–	–	10 / 8 / 8 / 9
MELD-Na score **	16 (10 – 21)	10 (9 – 16)	22 (18 – 28)
CHILD PUGH score **	9 (8 – 11)	8 (6 – 10)	10 (9 – 11)
Leukocytes count * (x 10^9^/L)	7.0 (4.7 – 9.5)	5.6 (4.3 – 7.4)	8.1 (4.7 – 12.3)
Lymphocytes count (x 10^9^/L)	1.1 (0.8 – 1.7)	1.1 (0.7 – 1.8)	1.1 (0.8 – 1.6)
Monocytes count * (x 10^9^/L)	0.7 (0.5 – 1.0)	0.6 (0.4 – 0.8)	0.9 (0.6 – 1.2)
Neutrophils count * (x 10^9^/L)	4.6 (3.1 – 6.8)	3.8 (2.8 – 5.4)	5.5 (3.1 – 8.5)
CRP * (mg/L)	20.2 (7.4 – 35.6)	5.9 (4.8 – 21.7)	24.7 (16.4 – 44)

1 Data are presented as median values (1st – 3rd quartile).

2 Median age of healthy control group (*n* = 27) was 39 (31–52) years old). Mann Whitney U Test: * *p* < 0.05 between CLD and AD; ** *p* < 0.0001 between CLD and AD.

### 2.2 Ethics approval

The study was approved by local research ethic committees (12/LO/0167). Informed consent was obtained by the next of kin if patients were not able to provide consent.

### 2.3 Isolation of mononuclear and polymophonuclear cells

Peripheral blood mononuclear cells (PBMCs), ascites mononuclear cells (AMNCs), fresh PMNs and monocytes were isolated as described in Supporting Methods.

### 2.4 Phenotyping and intracellular staining

CD8^+^ *T* cells were phenotyped using cell surface and intracellular staining as described in Supporting Methods. Multicolor flow cytometry analyses were performed on LSRFortessa™ flow cytometer, data were acquired using BD FACSDiva™ software (Becton Dickinson Ltd, Oxford, UK) and analyses were performed using FlowLogic software (Inivai Technologies, Pty Ltd).

### 2.5 Immunohistochemistry

Explanted liver tissue was obtained from patients undergoing orthotopic liver transplantation (OLT) for cirrhosis (alcoholic liver disease (ALD); *n* = 4 and non-alcoholic steatohepatitis (NASH); *n* = 4). Hepatic resection margins of colorectal metastases (*n* = 4) served as pathological controls. Double epitope immunohistochemistry (CD8 & HLA-DR) was performed for all cases followed by analysis using the Nuance multispectral imaging system (Perkin Elmer, Beaconsfield, UK) [12] (Supporting Methods).

### 2.6 Transcriptional analyses

**Fig. S1:**
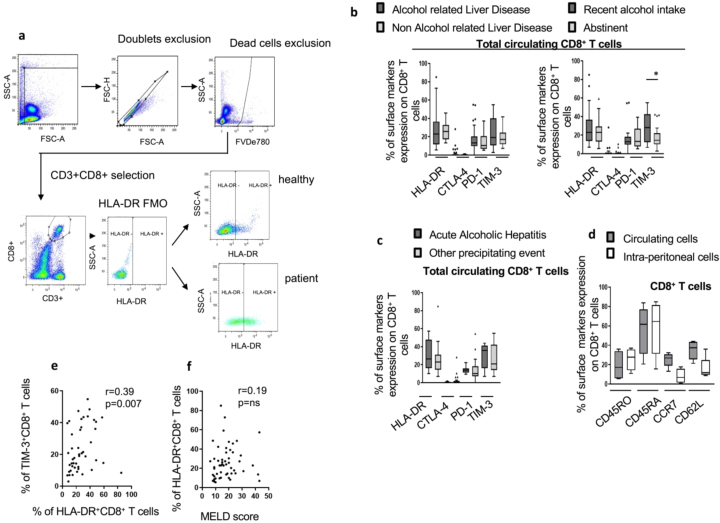
CD8+ T cells phenotyping using flow cytometry. (a) Gating/ cell sorting strategy to determine CD3+CD8+ T cells phenotype in cirrhotic patients and HV: frozen isolated PBMCs or AMNCs were thawed, stained by Fluorescence Viability Dye (FVD) and finally extra- or intra-cellular stained. Following doublets and live/dead exclusion, CD3/CD8 double positive cells were selected. The expression of extracellular or intracellular markers was then assessed on the CD3+CD8+ subset using Fluorescence Minus One (FMO) controls; (b) Phenotype of circulating CD8+ T cells in the entire cohort of cirrhotic patients according to history of alcohol consumption: total CD8+ T cells phenotype in patients with alcohol related liver disease (ALD) (n = 47) versus non-ALD patients (n = 13) (left panel) and in abstinent (n = 32) versus non-abstinent patients (n = 28) (right panel). (c) Phenotype of total circulating CD8+ T cells in AD patients according to the precipitating event: acute alcoholic hepatitis (AAH) (n = 10) versus other (n = 25). (d) Expression of maturation surface markers in peritoneal CD8+ T cells compared to paired circulating CD8+ T cells in cirrhotic patients (n = 7). (e) Correlations between TIM-3+CD8+ T cells and HLA-DR+CD8+ T cells (Spearman correlation; n = 46). (f) No correlations were detected between HLA-DR expression on CD8+ T cells and MELD score (n = 53)* p<0.05; ** p<0.0001 (Mann-Whitney U test (b;c); Wilcoxon signed-rank test (d); Spearman correlation (e;f)).

Viable CD3^+^CD8^+^ *T* cells were sorted into CD3^+^CD8^+^HLA-DR^+^ or CD3^+^CD8^+^HLA-DR^−^ using FACS Aria II flow cytometer (Becton Dickinson Ltd, Oxford, UK) (gating strategy described in Fig. S1a). NanoString nCounter® Immunology v2 Panel (NanoString™, Seattle, USA) profiling 579 genes involved in immune response was then performed and gene expression was compared in paired CD8^+^HLA-DR^−^ and CD8^+^HLA-DR^+^ *T* cells from HV (*n* = 3) or patients (*n* = 3). Analyses were carried out using the nSolver™ Analysis Software 3.0. Genes were identified according to gene ontology annotation.

### 2.7 Cell stimulation and measurement of cytokines production

HLA-DR^+^CD8^+^ *T* cells were purified using a multi-step magnetic cell separation isolation process (Supporting Methods). HLA-DR^−^ or HLA-DR^+^ CD8^+^ *T* cell subsets from HV or patients with cirrhosis were stimulated for 2 days with Dynabeads® human T-activator CD3/CD28 (Life Technologies Limited, Paisley, UK) at 1:1 bead to cell ratio. Supernatants were collected and assessed for cytokines production using a Multiplex Cytokines Detection System V-Plex Proinflammatory Panel 1 kit (Meso Scale Discovery (MSD), Rockville, USA) according to the manufacturer's instructions.

### 2.8 Assessment of CD8^+^*T* cells regulatory effect on autologous PBMCs proliferation

Total PBMCs from HV or patients with cirrhosis were cultured for 3 days either alone or in presence of pre-isolated autologous HLA-DR^−^ or HLA-DR^+^CD8^+^ *T* cells (1:2 T cell to PBMCs ratio) in the presence of CD3/CD28 beads (1:4 bead to PBMCs ratio). Proliferation of responders PBMCs was measured using carboxyfluorescein succinimidyl ester (CFSE) labelling (Life Technologies Limited, Paisley, UK) in the presence or absence of anti-CTLA-4, anti-PD-1 (Life Technologies Limited, Paisley, UK) or anti-TIM3 (Biolegend, California, USA) blocking antibodies (all used at 10 μg/ml). Cytokines production in blocking experiment was assessed using MSD V-Plex Proinflammatory Panel 1 kit (MSD, Rockville, USA).

### 2.9 Phenotypic assessment of myeloid cells following co-culture with HLA-DR^+^CD8^+^*T* cells

CD8^+^ *T* cells pre-conditioned in plasma from cirrhotic patients (Supporting Methods) were co-cultured with bead-isolated neutrophils or monocytes at a 1:2 T cell to myeloid cell ratio in the presence of anti-CD3 (0.5 μg/mL) (Life Technologies Limited, Paisley, UK). Following 24hr co-cultures, neutrophils or monocytes were assessed for phenotypic expression of CD16, CD64, CD11b, CD62L, CD66b and CD177 cell surface markers on neutrophils or PD1, CD163, HLA-DR, CD14 and MerTK on monocytes.

### 2.10 Assessment of neutrophils phagocytic capacity and cytokine production following co-culture with HLA-DR^+^CD8^+^*T* cells

Neutrophils were assessed for their ability to phagocytize *E. coli* (*E. coli*) following 4 hrs co-culture with HLA-DR^+^CD8^+^ *T* cells using pHrodo™ Red *E. coli* BioParticles (Lifetechnologies, UK) following the manufacturer's protocol. Levels of TNF-α and IL-8 expression in neutrophils in response to *E. coli* lipopolysaccharide (LPS) stimulation (100 ng/mL) were measured using intracellular cytokine staining.

### 2.11 Statistical analysis

Mann-Whitney test was used for nonparametric data. Wilcoxon matched-pairs signed rank test and paired t-test were used for paired tests. Spearman correlation coefficients were calculated. Gene expression is reported as fold change of detected mRNA expression levels normalised to baseline values observed in HLA-DR^−^CD8^+^. Statistical significance was assumed for P values ≤ 0.05. Data analysis was performed using GraphPad Prism 5 (GraphPad Software Inc., San Diego, CA).

## 3 Results

### 3.1 Clinical characteristics

Twenty five stable cirrhotic patients (CLD) and thirty five patients admitted for decompensation of cirrhosis (AD) were recruited into this study. The median age of the cohort was 54.6 years old, 60% were male, principally suffering of ALD (Table 1). AAH was the precipitating factor for 10 AD patients (Table 1). Median MELD score was 18 in the overall cohort and significantly increased with acute decompensation (Table 1). Leukocytes, monocytes and neutrophils counts as well as C-Reactive Protein levels in patients with AD were significantly higher compared to patients with CLD (Table 1).

### 3.2 Peripheral CD8^+^*T* cells from cirrhotic patients display an altered phenotype

**Fig 1:**
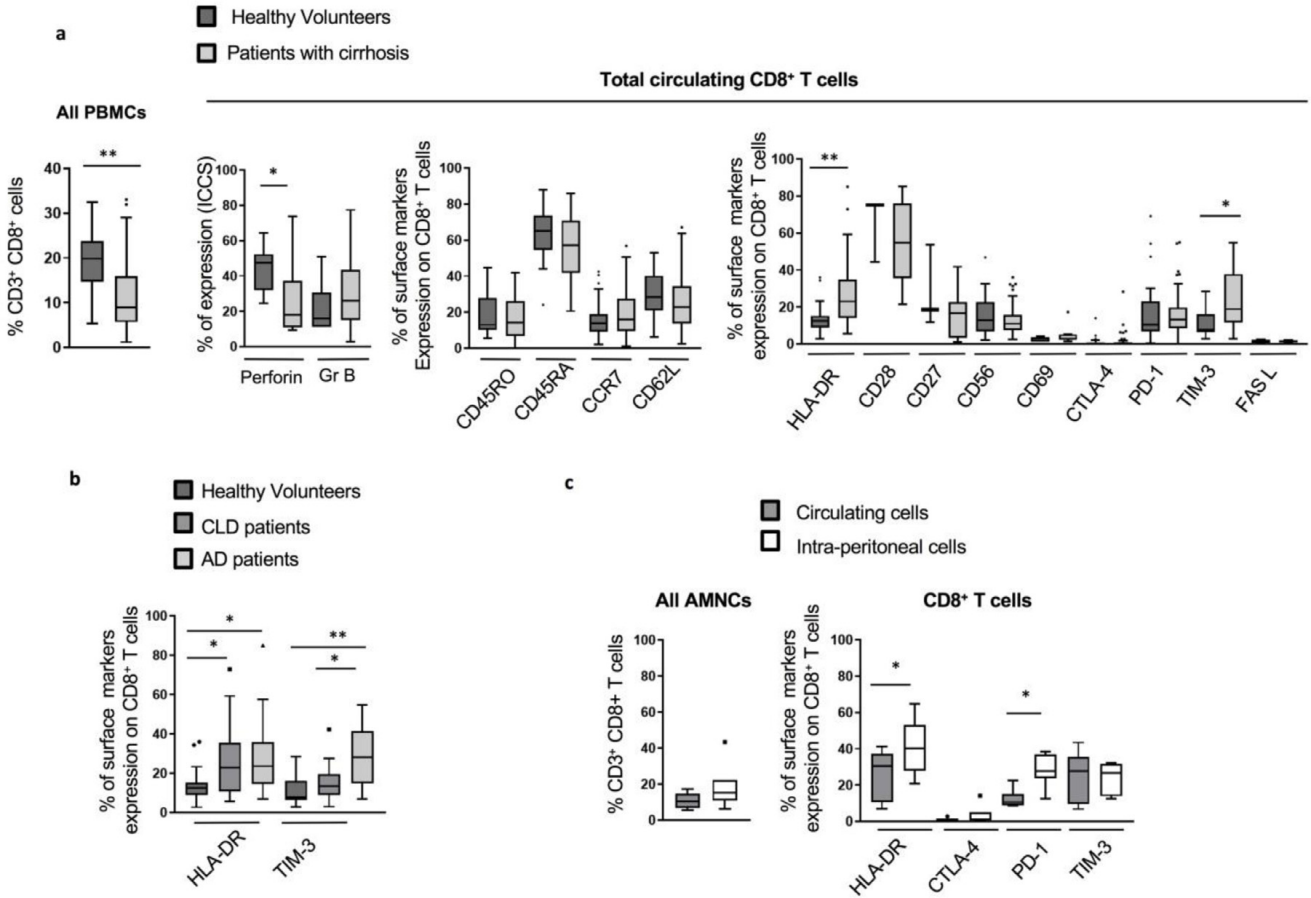
Phenotype of total CD8^+^*T* cells in cirrhotic patients assessed by flow cytometry. (a) Lower proportion and altered phenotype of total circulating CD8^+^*T* cells in cirrhotic patients (*n* = 60) compared to total circulating CD8^+^*T* cells in healthy volunteers (HV) (*n* = 27): proportion from peripheral blood mononuclear cells (PBMCs) (left panel), baseline intracellular functional markers (middle left panel), expression of maturation surface markers (middle right panel), activation and inhibition surface markers (right panel) were assessed by extra- or intra-cellular staining following live / dead exclusion. (b) Phenotype of total circulating CD8^+^*T* cells in cirrhotic patients according to disease severity: comparison of circulating CD8^+^*T* cells phenotype in HV (*n* = 27), stable chronic cirrhotic patients (chronic liver diseases (CLD)) (*n* = 25) and acute decompensated (AD) patients (*n* = 35). (c) Intra-peritoneal total CD8^+^*T* cells (isolated from ascites of cirrhotic patients, *n* = 7): proportion of CD8^+^*T* cells from ascites mononuclear cells (AMNCs) comparde to paired peripheral blood (left panel), percentage of expression of activation and inhibition surface markers (right panel). * *p*<0.05; ** *p*<0.0001 (Mann-Whitney U test (A; B) and Wilcoxon signed-rank test (C)).

In line with previous reports [5], [14], [18], the proportion of circulating CD8^+^ *T* cells in cirrhotic patients was significantly diminished compared to HV (median values: 8.99% *vs* 19.85% respectively; *p*< 0.0001). Furthermore, analyses of cytolytic mediators in CD8^+^ *T* cells from patients with cirrhosis showed significantly decreased perforin expression compared to HV (median values 18.05% *vs* 47.53%, respectively; *p* = 0.049) (Fig. 1a). No significant changes were detected in intracellular granzyme B levels or in the surface expression of T cell markers (CD45RO, CD45RA, CCR7 and CD62L) (Fig. 1a). T cell activation and inhibition markers however were markedly elevated in patients with cirrhosis (HLA-DR; *p*<0.0001 and TIM-3; *p* = 0.0002) in comparison to HV (Fig. 1a). The increase in HLA-DR expression on CD8^+^ *T* cells in cirrhosis was independent of disease severity (Fig. 1b). Conversely, higher levels of TIM-3 were particularly noted in patients with AD (Fig. 1b). This phenotype was not altered by disease aetiology (ALD *vs* non-ALD) or recent alcohol intake, with the exception of TIM-3 expression which was more pronounced in patients with recent alcohol consumption (Fig. S1b). AAH as a precipitating factor for AD did not affect the reported CD8^+^ *T* cell phenotype (Fig. S1c).

### 3.3 Peritoneal CD8^+^*T* cells share similar characteristics with matched peripheral CD8^+^*T* cells in cirrhotic patients with ascites

Expression of immune phenotypic markers on CD8^+^ *T* cells from cirrhotic patients with ascites, without spontaneous bacterial complications, was assessed and compared to paired peripheral blood CD8^+^ *T* cells. Although similar in proportion, peritoneal CD8^+^ *T* cells expressed greater levels of HLA-DR (*p* = 0.03) (Fig. 1c). Additionally, inhibitory receptor PD-1 was significantly up-regulated on peritoneal compared to peripheral CD8^+^ *T* cells (*p* = 0.01) (Fig. 1c). No significant differences were detected in the expression of other T cell markers (Fig. S1d).

### 3.4 HLA-DR^+^CD8^+^*T* cells are enriched in the liver of cirrhotic patients

**Fig 2:**
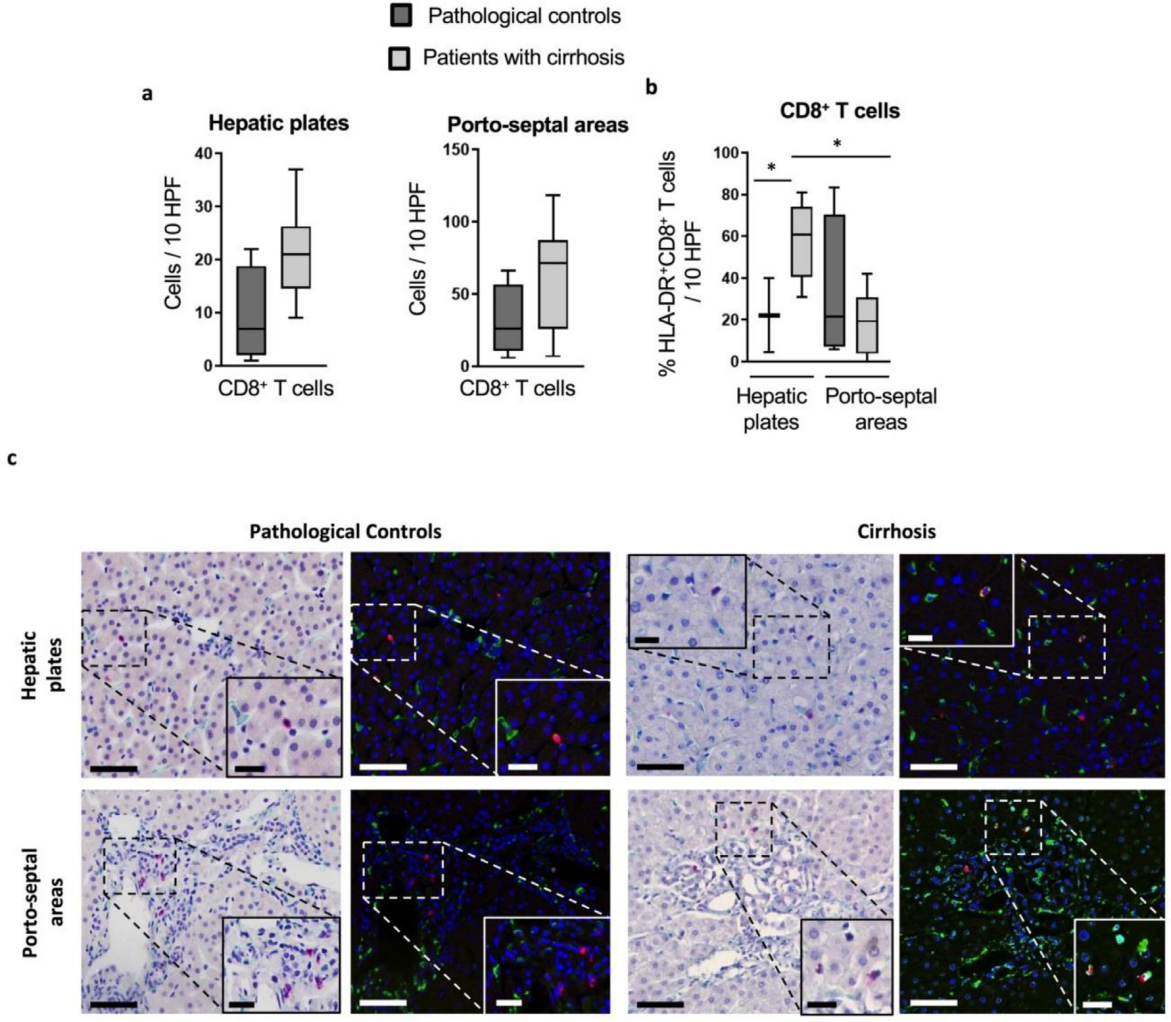
Characterization of intrahepatic CD8^+^*T* cells in cirrhotic patients and pathological controls: Immunohistochemistry was performed on formalin-fixed and paraffin-embedded explanted liver tissue from cirrhotic patients (*n* = 8) and hepatic resection margins of colorectal metastases as pathological controls (*n* = 4). Micrographs were analyzed using the Nuance multispectral imaging system to obtain (a) the number of CD8^+^*T* cells/10 random high-power fields (HPF) in hepatic plates (left panel) and porto-septal areas (right panel), and (b) the proportion of HLA-DR^+^ cells within the intrahepatic CD8^+^*T* cell contingent per 10 random HPF.* *p*< 0.05. (c) Representative micrographs for CD8/HLA-DR double epitope enzymatic immunohistochemistry in pathological controls (left panel) and cirrhotic livers (right panel) (400x); left columns: bright field micrographs showing CD8^+^ cells in red, and HLA-DR^+^ cells in green, with hematoxylin as nuclear counterstaining; right columns: corresponding pseudo-fluorescent micrographs showing CD8^+^ cells in red, HLA-DR^+^ cells in green, nuclei in blue, and co-localization of CD8 and HLA-DR signals in yellow. (Scale bars: 50 μm for 400x magnification; 20 μm for insets). (Man-Whitney U test for comparison between cirrhotic patients and pathological controls; Wilcoxon signed-rank test for comparison between hepatic plates and porto-septal areas).

Considering our results showing pronounced HLA-DR expression of peripheral and peritoneal CD8^+^ *T* cells, we tested whether CD8^+^ *T* cells in the liver featured similar characteristics. We reveal that HLA-DR^+^CD8^+^ *T* cells were enriched in liver explants from cirrhotic patients (ALD; *n* = 4 and NASH; *n* = 4). Despite no changes in the overall CD8^+^ *T* cells number, immunohistochemistry revealed a significantly higher proportion of HLA-DR^+^CD8^+^ *T* cells in cirrhotic liver tissues compared to non-cirrhotic pathological controls (median 60.75% *vs* 22.2%, respectively; *p* = 0.048) (Fig. 2a and b). In the explants from patients with cirrhosis, HLA-DR^+^CD8^+^ *T* cells were more enriched in the hepatic plates than in the porto-septal areas (median 60.75% *vs* 19.32% respectively; *p* = 0.001) (Fig. 2b and 2c). No difference was reported according to disease aetiology (data not shown).

### 3.5 Correlation between elevated markers, disease severity indices and susceptibility to infections in CLD

**Fig 3:**
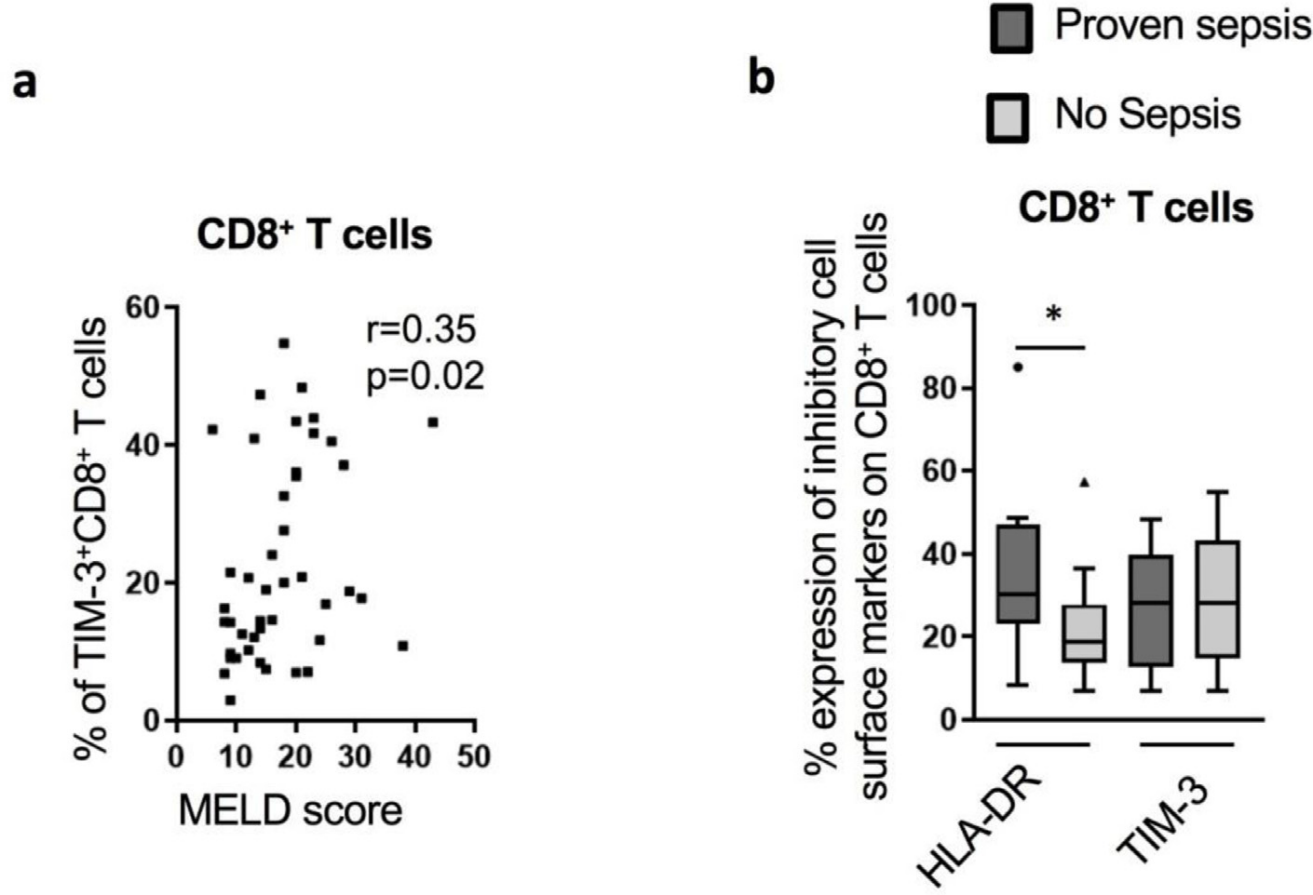
CD8^+^*T* cells phenotype in cirrhotic patients and association with disease severity and infection. (a) TIM-3 expression on CD8^+^*T* cells surface correlated positively with MELD score (*n* = 44). (b) The proportion of HLA-DR^+^CD8^+^*T* cells in AD (*n* = 35) was increased in patients with concomitant proven sepsis (*n* = 13) compared to non-septic patients (*n* = 22). * *p*<0.05 (Spearman correlation (a) and Mann-Whitney U test (b)).

When assessed for correlation with disease severity indices, elevated levels of TIM-3 on total CD8^+^ *T* cells correlated positively with MELD score (*r* = 0.35; *p* = 0.02) (Fig. 3a). We reveal a significant association between the proportion of TIM-3^+^CD8^+^ *T* cells and HLA-DR^+^CD8^+^ *T* cells (Fig. S1e). Expression of HLA-DR on CD8^+^ *T* cells did not correlate with MELD score (Fig. S1f). However, HLA-DR^+^CD8^+^ *T* cells were significantly higher in patients with AD presenting with concomitant sepsis at admission (Fig. 3b).

### 3.6 Characterization of the expanded HLA-DR^+^CD8^+^*T* cell population in cirrhotic patients and association with poor outcomes

**Fig 4:**
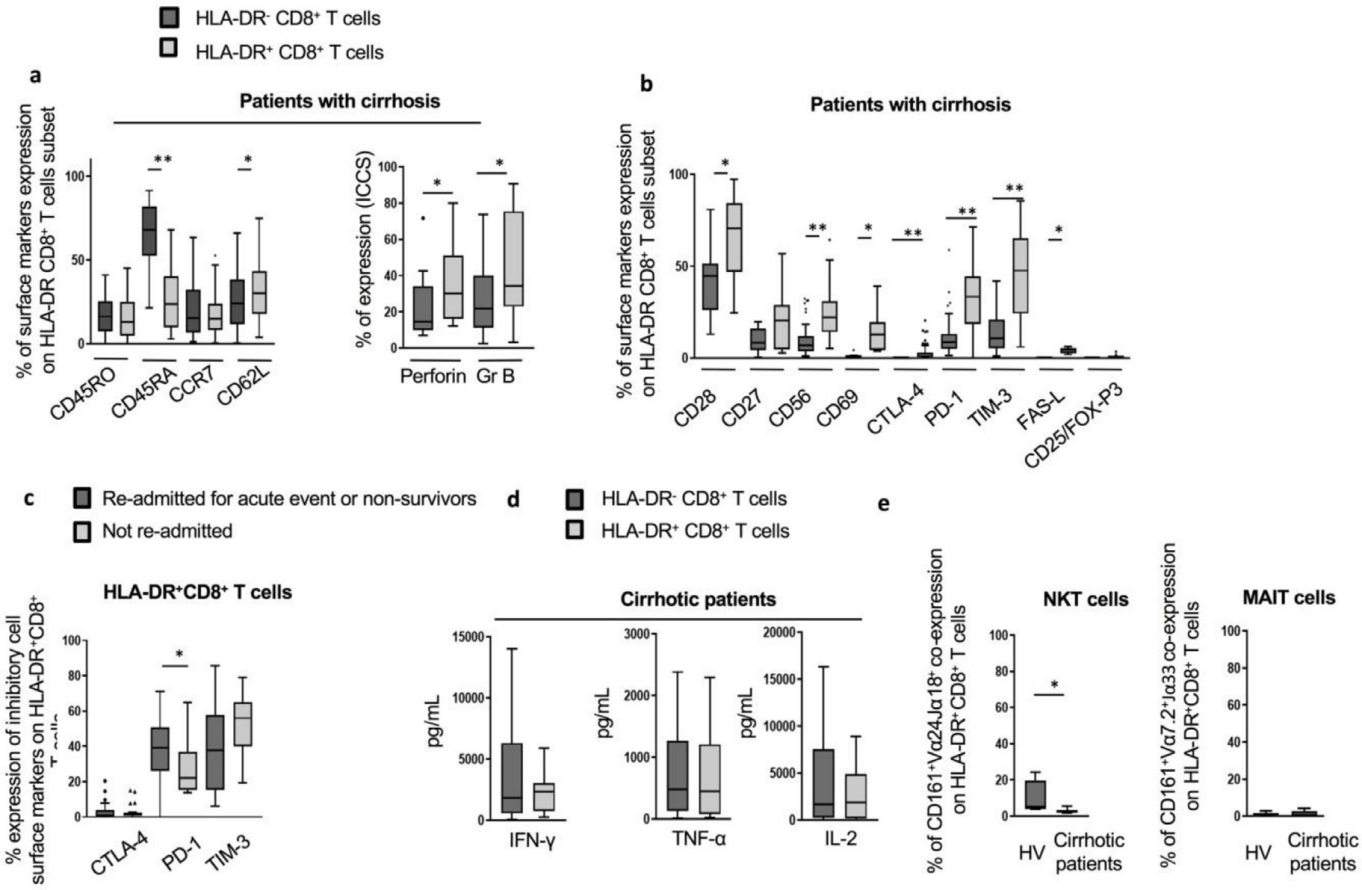
Detailed phenotypic and functional characterization of HLA-DR^+^CD8^+^*T* cells in cirrhotic patients. (a) Expression of maturation surface markers (left panel; *n* = 60) and baseline intracellular functional markers (right panel) in HLA-DR^−^CD8^+^*T* cells compared to HLA-DR^+^CD8^+^*T* cells. (b) Expression of activation and inhibition surface markers, including expression of T-regulator cells markers (CD25 and FOXP-3). (c) Elevated frequency of CD8^+^*T* cells co-expressing PD1 and HLA-DR in patients with poor outcomes. (d) *In vitro* cytokines production of HLA-DR^−^CD8^+^ and HLA-DR^+^CD8^+^*T* cells isolated from cirrhotic patients following 48 h CD3/CD28 beads stimulation (*n* = 10). (e) Co-expression of natural killer T (NKT) (left panel) and mucosal-associated invariant T (MAIT) cell markers (right panel) on HLA-DR^+^CD8^+^*T* cells from HV (*n* = 4 and *n* = 9, respectively) and cirrhotic patients (*n* = 10 and *n* = 18 respectively). * *p*<0.05; ** *p*<0.0001 (Wilcoxon signed-rank test (a; b; d; e) and Mann-Whitney U test (c)).

**Fig. S2:**
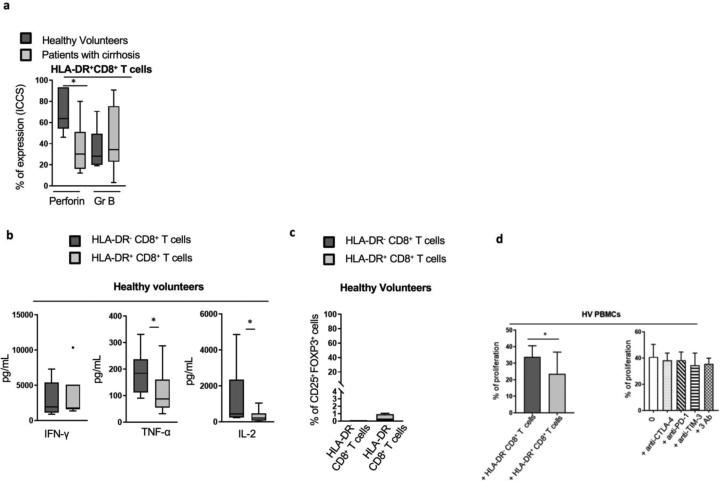
Phenotypic and functional characteristics of HLA-DR+CD8+ T cells isolated from HV. (a) Perforin and Granzyme B levels in HLA-DR+CD8+ T cells from HV (n = 6) and patients (n = 9). (b) In vitro cytokines production of HLA-DR- or HLA-DR+ CD8+ T cells from HV following 48h CD3/CD28 beads stimulation (n = 8). (c) Expression of classical T-regulator cells markers in HLA-DR+CD8+ T cells and paired HLA-DR-CD8+ T cells from HV (n = 5). (d) Proliferation of autologous PBMCs following co-culture in the presence of HLA-DR+ or HLA-DR-CD8+ T cells from HV (n = 7) (1:2 HLA-DR+ or HLA-DR- CD8+ T cell to PBMCs ratio) (left panel). The effect of checkpoint receptor blockade was assessed in the presence of HLA-DR+CD8+ T cells (n = 3) (right panel). * p<0.05 * p<0.05; ** p<0.0001 (Mann-Whitney U test (a); Wilcoxon signed-rank test (b;c;d))

Subsequent detailed analyses of the HLA-DR^+^CD8^+^ *T* cell subset were carried out in cirrhotic patients and revealed that when compared to their HLA-DR^−^ counterparts, these cells exhibited a T central memory (Tcm) phenotype highlighted by low expression of the naïve T cell marker CD45RA (23.80% *vs* 67.89%, *p*<0.0001) and high levels of the lymph node homing marker, CD62L (Fig. 4a). We also observed significantly enhanced levels of perforin and granzyme B in HLA-DR^+^CD8^+^ *T* cells compared to paired HLA-DR^−^CD8^+^ *T* cells from cirrhotic patients (*p* = 0.012) (Fig. 4a). However, perforin levels in HLA-DR^+^CD8^+^ *T* cells from patients remained significantly lower in comparison to the levels seen in HLA-DR^+^CD8^+^ *T* cells from HV (Fig. S2a). Similarly, HLA-DR^+^CD8^+^ *T* cells from cirrhotic patients displayed high levels of activation markers (CD28, CD56 and CD69) and significant increase in inhibitory markers CTLA-4 (*p*<0.0001), PD-1 (*p*<0.0001), TIM-3 (*p*<0.0001) and the effector marker FAS-L (*p* = 0.002) (Fig. 4b). HLA-DR^+^CD8^+^ *T* cells expressing high levels of PD-1 were associated with poor disease outcomes (Fig. 4c).

Functional assessment following CD3/CD28 beads stimulation revealed no differences in secreted levels of IFN-γ, TNF-α or IL-2 in paired HLA-DR^+^ *versus* their HLA-DR^−^CD8^+^ counterparts from cirrhotic patients (Fig. 4d). However, HLA-DR^+^CD8^+^ *T* cells from HV produced significantly lower levels of TNF-α (*p* = 0.02) and IL-2 (*p* = 0.008) (Fig. S2b).

### 3.7 HLA-DR^+^CD8^+^*T* cells in patients with cirrhosis differ from classical regulatory, NKT and MAIT cells

In line with previous reports [16], [19], levels of CD25^+^FOX-P3^+^ double positivity in the HLA-DR^+^CD8^+^ *T* cell population were negligible in cirrhotic patients as well as in HV (Fig. 4b and Fig. S2c). Despite high levels of markers known not to be restricted to CD8^+^ *T* cells, HLA-DR^+^CD8^+^ *T* cells lack the expression of invariant TCR receptors Vα24Jα18 and Vα7.2Jα33, distinguishing them from NKT and MAIT cells, respectively (Fig. 4e).

### 3.8 Transcriptional analyses reveal an impairment of pro-inflammatory pathways in HLA-DR^+^CD8^+^*T* cells from cirrhotic patients

**Fig 5:**
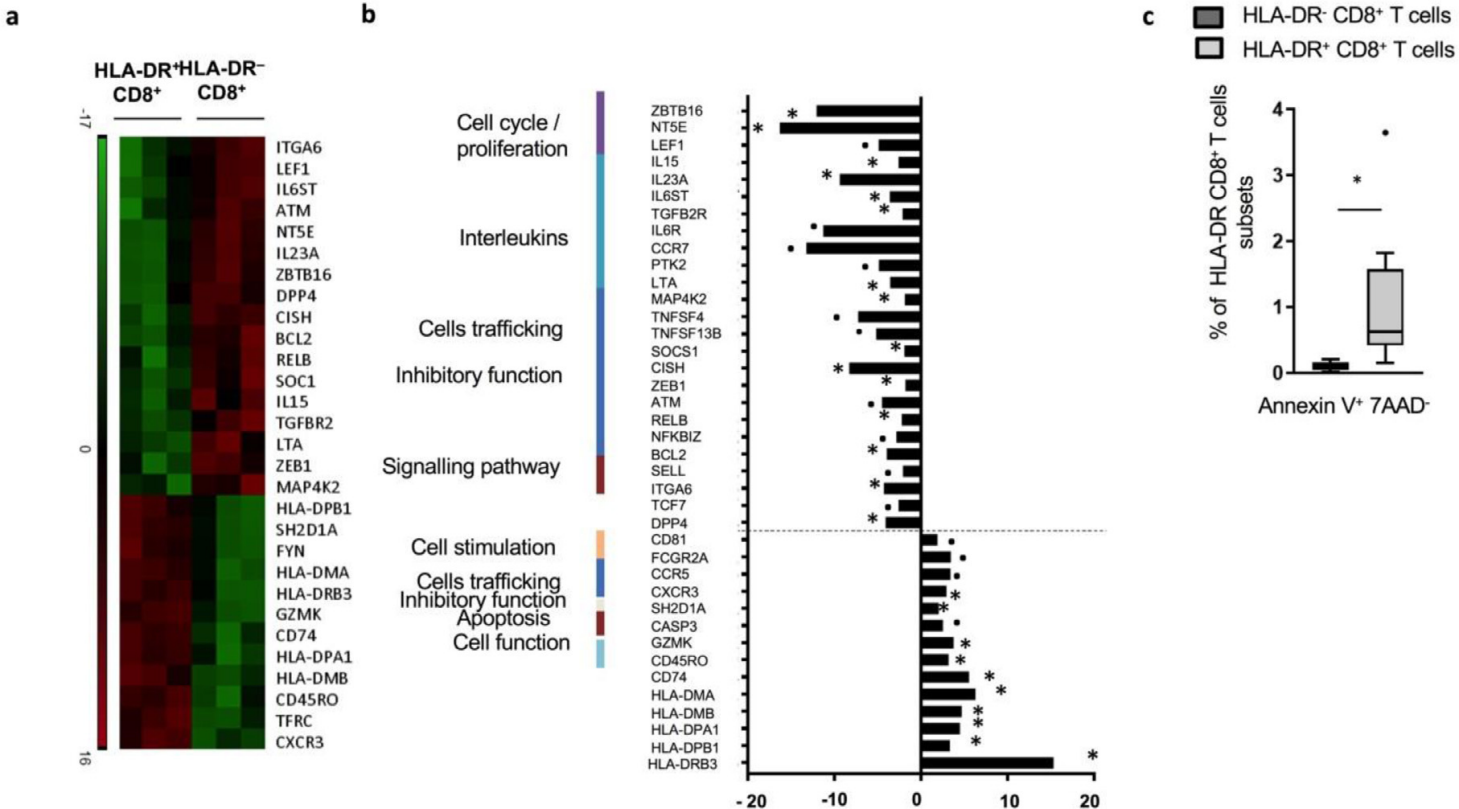
Transcriptional features of HLA-DR^+^CD8^+^*T* cells from cirrhotic patients. Following live/ dead exclusion, flow cytometry sorted cells were assessed for the expression of 579 genes involved in immune responses using Nanostring™ technology. Gene expression is reported in HLA-DR^+^CD8^+^*T* cells from cirrhotic patients as fold change of detected mRNA expression levels normalised to baseline values observed in HLA-DR^−^CD8^+^ paired samples (*n* = 3, CLD patients; *n* = 2, AD patients; *n* = 1)): (a) Heat-map created using Java TreeView 1.1.6r4 representing statistically significant transcriptional changes between HLA-DR^+^ and HLA-DR^−^ CD8^+^*T* cells in cirrhotic patients (*n* = 3). (b) Relevant transcriptional changes between HLA-DR^+^ and HLA-DR^−^ CD8^+^*T* cells in cirrhotic patients. (c) *Ex-vivo* apoptosis staining performed to explore levels of apoptosis in HLA-DR^+^ and HLA-DR^−^CD8^+^*T* cells from cirrhotic patients (*n* = 8). * *p*<0.05; **•***p*<0.1 (Wilcoxon signed-rank test).

**Fig. S3:**
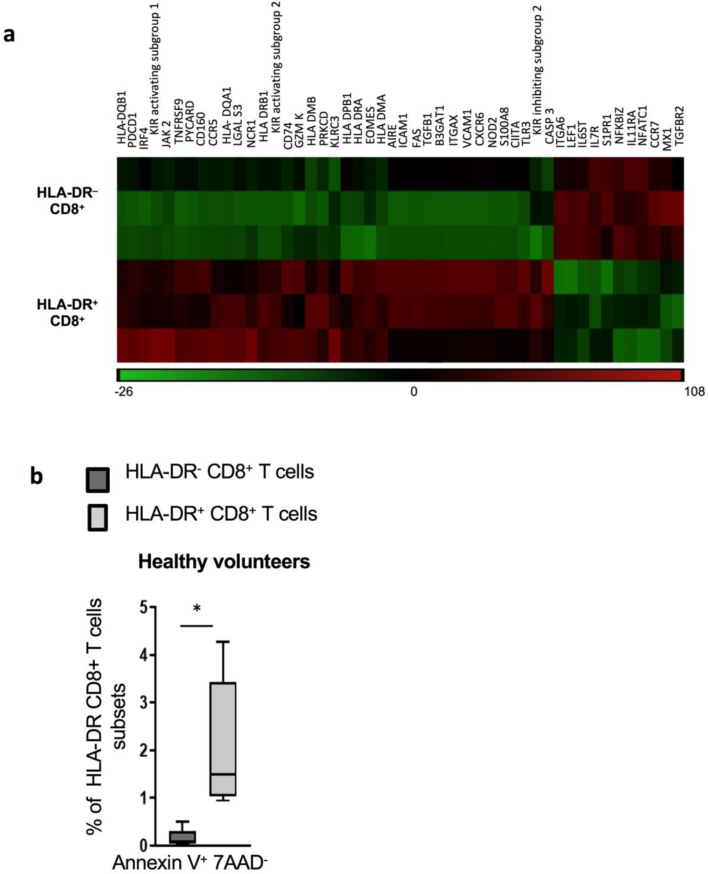
Phenotypic and functional characteristics of HLA-DR+CD8+ T cells isolated from HV. (a) Perforin and Granzyme B levels in HLA-DR+CD8+ T cells from HV (n = 6) and patients (n = 9). (b) In vitro cytokines production of HLA-DR- or HLA-DR+ CD8+ T cells from HV following 48h CD3/CD28 beads stimulation (n = 8). (c) Expression of classical T-regulator cells markers in HLA-DR+CD8+ T cells and paired HLA-DR-CD8+ T cells from HV (n = 5). (d) Proliferation of autologous PBMCs following co-culture in the presence of HLA-DR+ or HLA-DR-CD8+ T cells from HV (n = 7) (1:2 HLA-DR+ or HLA-DR- CD8+ T cell to PBMCs ratio) (left panel). The effect of checkpoint receptor blockade was assessed in the presence of HLA-DR+CD8+ T cells (n = 3) (right panel). * p<0.05 * p<0.05; ** p<0.0001 (Mann-Whitney U test (a); Wilcoxon signed-rank test (b;c;d))

Nanostring transcriptional analysis revealed 29 significantly differentially expressed genes between paired HLA-DR^+^ and their HLA-DR^−^ counterparts in cirrhotic patients (Fig. 5a and 5b). Besides the significant up-regulation of genes coding for HLA proteins in the HLA-DR^+^ subset, we report an up-regulation of granzyme K (*GZMK*) coding gene. Conversely, levels of genes involved in cytokine production and intracellular signalling (*IL-15, IL-23A, IL6-ST, LTA, TGFB2R*), genes involved in JAK/STAT (*CISH, SOCS1*) and NFκB pathways (*RELB, BCL2, ATM*) were diminished. Transcriptomic analysis also suggested impairment in the TNF-α signalling pathway with a significant down-regulation of *LTA, IL15, MAP4K2* and a trend to a decreased expression of *TNFSF4, TNFSF13B* (Fig. 5a and 5b). Similarly, significant alteration in expression of genes associated with TNF-α signalling pathways, cytokines production and signalling were noted in the HLA-DR^+^ subset from HV (Fig. S3a).

### 3.9 HLA-DR^+^CD8^+^*T* cells display low levels of apoptosis

Having defined phenotypic and transcriptomic alterations, we then assessed levels of apoptosis in HLA-DR^+^CD8^+^ *T* cells. Despite a higher rate of Annexin^+^ 7-AAD^–^ cells in the HLA-DR^+^ subset from patients compared to paired HLA-DR^−^, the proportions remained subtle (median 0.68% and 0.10%; respectively, *p* = 0.008) (Fig. 5c). This was also the case in the HLA-DR^+^ cells from HV (Fig. S3b).

### 3.10 HLA-DR^+^CD8^+^*T* cells from cirrhotic patients show a diminished capacity to induce proliferation of autologous PBMCs

**Fig 6:**
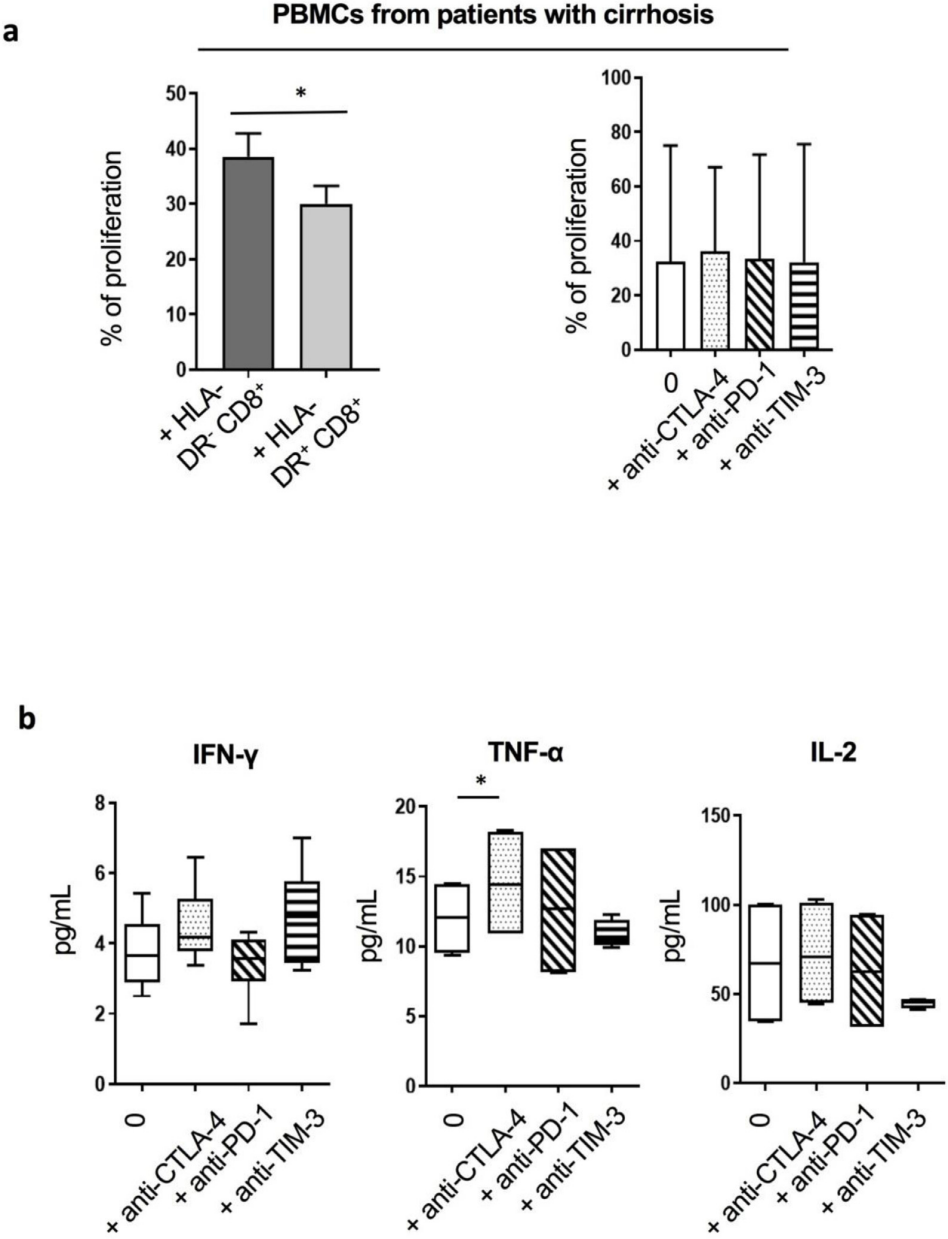
HLA-DR^+^CD8^+^*T* cells are less potent at inducing autologous PBMCs’ proliferation. (a) Proliferation *in vitro* of total autologous PBMCs in the presence of pre-isolated HLA-DR^+^ or HLA-DR^−^CD8^+^*T* cells from cirrhotic patients (*n* = 7; CLD patients; *n* = 3, AD patients; *n* = 4) (1:2 HLA-DR^+^ or HLA-DR^−^ CD8^+^*T* cell to PBMCs ratio) (left panel). The impact of surface inhibitory receptors blockade to restore the proliferation was tested in the presence of HLA-DR^+^CD8^+^*T* cells and autologous PBMCs from cirrhotic patients (total *n* = 6; AD patients; *n* = 3, CLD patients; *n* = 3) (right panel). (b) Inhibitory receptor blockade was followed by assessment of cytokines production (*n* = 4). * *p*<0.05 (Wilcoxon signed-rank test (a), Paired t-test (b)).

Functional assay to test the effect of HLA-DR^+^CD8^+^ *T* cells on the proliferation of autologous CFSE-stained PBMCs *in vitro* showed that HLA-DR^+^CD8^+^ *T* cells from cirrhotic patients induced lower levels of total PBMC proliferation in comparison to their HLA-DR^−^ counterparts (Fig. 6a). Blockade of the immune checkpoints CTLA-4, PD-1 and TIM-3 failed to improve this proliferation (Fig. 6a). Similarly, proliferation of PBMCs from HV was significantly impaired in the presence of autologous HLA-DR^+^CD8^+^ *T* cells and was not reversed following blockade of the inhibitory markers (Fig. S2d). In contrast, CTLA-4 blockade resulted in a significant increase of TNF-α in co-cultures of autologous PBMCs with HLA-DR^+^CD8^+^ *T* cells from cirrhotic patients (Fig. 6b).

### 3.11 *In vitro*, HLA-DR^+^CD8^+^*T* cells from patients alter the phenotype and function of autologous myeloid cells

**Fig 7:**
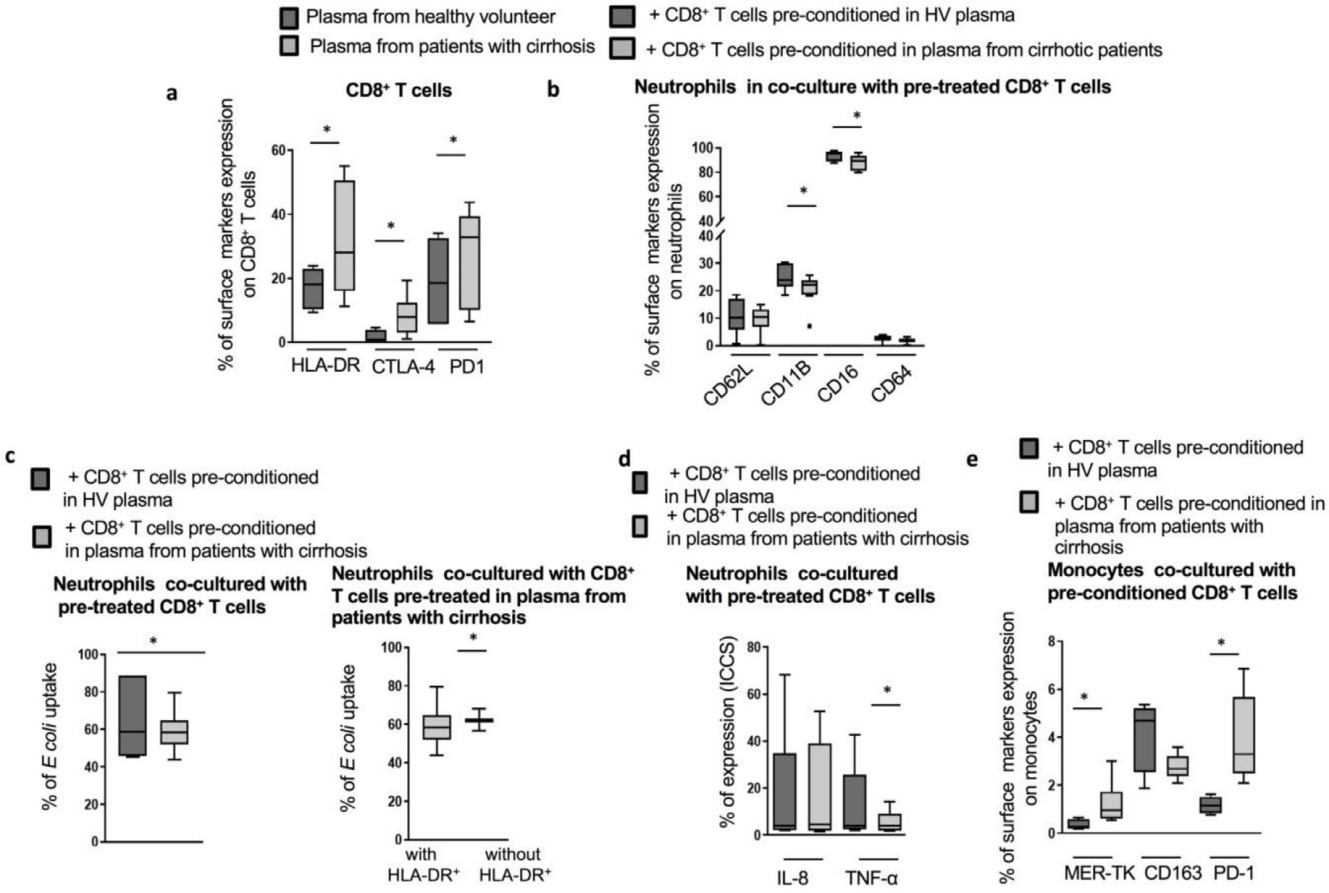
Interaction between HLA-DR^+^CD8^+^*T* cells and autologous neutrophils and monocytes (a) CD8^+^*T* from HV cultured for 3 days in the presence of 25% plasma derived from AD patients (*n* = 8) induces a similar phenotype to the *ex vivo* CD8^+^*T* cells from cirrhotic patients contrary to HV CD8^+^*T* cells cultured in the presence of 25% HV plasma (*n* = 10). (b) Phenotype of autologous fresh neutrophils following 24 hr co-culture with AD cirrhotic-plasma (HLA-DR^high^) *vs* HV-plasma (HLA-DR^low^) treated CD8^+^*T* cells. (c) Phagocytic ability of neutrophils following 4 h co-culture with HLA-DR^high^*vs* HLA-DR^low^ CD8^+^*T* cells (left panel) as well as following the removal of HLA-DR^high^ from total CD8^+^*T* cell population (right panel). (d) Cytokine productions in neutrophils following 4 h of co-culture. (e) Phenotypic changes on co-cultured monocytes with plasma pre-conditioned CD8^+^*T* cells (HV-plasma (*n* = 4) and AD cirrhotic patients plasma (*n* = 7)) at 24 h. * *p*<0.05. (Wilcoxon signed-rank test (a;b;d;e), Paired t-test (c)).

We investigated the impact of HLA-DR^+^CD8^+^ *T* cells on myeloid cells using an *in vitro* co-culture system. We demonstrated that following 72hr of culture in the presence of 25% of plasma derived from AD patients, isolated CD8^+^ *T* cells from HV had enhanced HLA-DR expression, mimicking the *ex vivo* phenotype (Fig. 7a). The conditioned cells were then co-cultured with autologous neutrophils or monocytes. Co-cultures with neutrophils resulted in a significantly lower expression of activation markers (CD16 and CD11B) (Fig. 7b). Additionally, the phagocytosis ability of the co-cultured neutrophils was significantly impaired but partially restored when the HLA-DR^+^ subset was removed prior to initiating co-culture (Fig. 7c). Following LPS stimulation, neutrophils co-cultured with HLA-DR^+^CD8^+^ *T* cells exhibited a significant decrease in their capacity to produce TNF-α (Fig. 7d). When co-cultured with HLA-DR^+^CD8^+^ *T* cells, monocytes displayed an immunosuppressive phenotype characterized by significantly increased levels of MER-TK and PD-1 expression (Fig. 7e).

## 4 Discussion

We report a detailed phenotypical and functional description of CD8^+^ *T* cells in cirrhosis revealing an expansion of an HLA-DR^+^CD8^+^ *T* cell subset in peripheral, peritoneal and intrahepatic compartments in this group of patients. Moreover, this HLA-DR^high^ phenotype showed elevated levels of inhibitory receptors (CTLA-4, PD-1 and TIM-3). Proportions and inhibitory profile of peripheral HLA-DR^+^CD8^+^ *T* cells were associated with infections and poor disease outcomes. Our findings provide transcriptional basis for the functional properties of the HLA-DR^+^CD8^+^ *T* cells isolated from cirrhotic patients. We also shed light on the interaction of this population with PBMCs and myeloid cells and their possible contribution to impaired innate immune responses in cirrhosis.

It is of significance that recent findings demonstrate the expansion of a similar phenotype in a cohort of patients with septic shock. Mouillaux et al. showed an expansion of PD1-co-expressing HLA-DR T cells with impaired function following TCR activation [20]. These data, described in another model of systemic immune dysfunction, reinforces our observations and add further emphasis to the role of TCR activation in the initiation of this phenotype and the induction of immune alterations in this population of CD8^+^ *T* cells.

Elevated surface expression of immune checkpoint molecules (PD-1, CTLA-4 and TIM-3) on the HLA-DR^+^CD8^+^ *T* cells was suggestive of an exhausted T cell profile. Recent observations however have referred to up-regulation of checkpoint receptors coupled with reduction in functions as a sign of functional adaptation to a chronically inflamed milieu rather than signs of T cell exhaustion [21]. Additionally, one of the distinguishing features between exhausted and “non-functional” cells is the reversibility and regain of functions following blockade of inhibitory receptors in the latter populations [22]. The data presented here suggest a role of CTLA-4 blockade in restoring TNF-α production. Previous studies have also shown that in the context of AAH, blockade of highly expressed surface inhibitory receptors PD-1 and TIM-3 on CD4^+^ and CD8^+^ *T* cells enabled the restoration of cytokines production [10]. As opposed to exhausted T cells which exhibit increased levels of apoptosis, susceptibility to apoptosis was not detected in activated HLA-DR^High^ T cells in cirrhosis.

Although well known for their antigen presentation capacity, MHC class II molecule expression on activated T cells have been shown to trigger a variety of signalling pathways inducing both apoptosis and clonal anergy leading to failure to respond to subsequent antigenic stimuli [23], [24]. When first described, naturally occurring HLA-DR^+^CD8^+^ regulatory T cells were postulated to expand following interaction with foreign antigens and their suppressive capacity was demonstrated to be CTLA-4-mediated [16]. In cirrhosis, bacterial translocation as well as bacterial products have been shown to be implicated in the impairment of innate immune function and could play a role in the induction of this phenotype. Furthermore, in view of the importance of the inflammatory microenvironment in modulating immune cell function and as proven by the mimicking of the *ex vivo* phenotype following *in vitro* exposure to patients sera, it is likely that this phenotype is also driven by soluble mediators in the circulation. The precise factors are still unclear and remain to be investigated. More studies are necessary to investigate the mechanisms underpinning the induction of HLA-DR expression on CD8^+^ *T* cells in cirrhosis.

Transcriptional analysis of HLA-DR^+^CD8^+^ *T* cells reflected the phenotypical changes and highlighted a global down-regulation in gene expression including genes involved in pro-inflammatory cytokines production and intracellular signalling (*IL-15, IL-23A, IL6-ST, LTA, TGFB2R*). Notably, TNF-α signalling pathways seemed impaired in HLA-DR^+^CD8^+^ *T* cells compared to HLA-DR^−^CD8^+^ *T* cells. With the exception of HLA-coding genes, much fewer genes (such as Granzyme K (*GZK*)) were up-regulated. *GZK* has been previously reported to be over-expressed in a particular subset of CD8^+^ *T* cells isolated by single-cell sequencing of liver cancer-infiltrating T cells where different clusters of infiltrating CD8^+^ *T* cells were identified according to transcriptional features and notably a *GZK*-expressing cluster sharing some exhausted transcriptional characteristics with uncommon activation markers but different from the typical exhausted CD8^+^ cluster [25]. Identification of expanded HLA-DR^+^CD8^+^ *T* cells in other chronic inflammatory conditions and full characterization of their gene expression profile are necessary before defining a gene expression signature specific to cirrhosis.

Despite the association between recent alcohol intake and TIM-3 expression on CD8^+^ *T* cells in the most severe group of patients (AD), it is worth noting that there were no other detectable differences in the phenotype of the CD8^+^ *T* cells based on disease aetiology. Correlations between TIM-3 expression in total CD8^+^ *T* cells and the severity of liver disease corroborated the link between disease stage and CD8^+^ *T* cells dysfunctional profile. Although no significant correlation between HLA-DR expression on CD8^+^ *T* cells and MELD score were detected, circulating HLA-DR^+^CD8^+^ *T* cells levels were higher in AD patients with concomitant sepsis suggesting a possible contribution of this population to susceptibility and/or impaired immune responses to infection episodes. Therefore, monitoring the expansion of this CD8^+^ *T* cell subset could serve as a potential immunomodulatory marker to facilitate the discrimination of cirrhotic patients with high risk of developing infection. Our finding that co-expression of HLA-DR and PD1 was a predictor of outcome highlights that it is more often a panel of biomarkers, as opposed to expression of a single molecule that is more likely to identify patients at high risk. However, this study only describes a cohort of 60 patients with cirrhosis; the clinical utility of this phenotype should be measured in a larger cohort of patients. Furthermore, our findings need to be validated in other cohorts of liver disease patients, stratifying for disease aetiology and using larger cohorts, followed over time.

In accordance with previous reports [16], [19], HLA-DR^+^CD8^+^ *T* cell subset reported in our study were immunomodulatory through their capacity to induce weaker PBMCs proliferation compared to their HLA-DR^−^ counterparts. Sera-conditioning experiments highlight the importance of the inflammatory milieu in driving the predominant HLA-DR^+^CD8^+^ *T* cells phenotype in cirrhosis. The role of the microenvironment in the induction of this phenotype may also explain the functional differences observed between HLA-DR^+^CD8^+^ *T* cells from cirrhotic patients and HV [16], [17].

In our study, although it failed to restore proliferation, CTLA-4 blockade enhanced levels of TNF-α production in PBMCs from cirrhotic patients. Although the interplay between CD8^+^ *T* cells and neutrophils have been previously demonstrated [26], our study is the first to report an immunomodulatory role of dysfunctional CD8^+^ *T* cells on PMNs in cirrhosis, particularly in regards to TNF-α secretion. Indeed, we reveal further immune-regulatory functions exerted by the HLA-DR^high^ population on monocytes. We demonstrate that HLA-DR^+^CD8^+^ *T* cells induced a regulatory monocyte phenotype highlighted by higher MER-TK and PD-1 expression. MER-TK^+^ monocytes have been reported to increase in ACLF patients, correlate with disease severity and to display impaired pro-inflammatory cytokines production [12]. Our *in vitro* data showing induced-PD-1 expression in monocytes however requires further studies to explore *ex vivo* expression levels of PD-1 on monocytes from patients with cirrhosis. These findings strongly support the hypothesis of an initial protective role of the dysfunctional CD8^+^ *T* cells in cirrhotic patients following an excessive inflammation, by inducing pro-resolution monocytes phenotype and lower activation status of neutrophils. This however may have deleterious effects on antimicrobial responses and contribute to subsequent impairment in immune functions and poor disease outcomes in these patients. This is further emphasized by the presence of this phenotype in the peritoneal compartment, which may contribute to susceptibility to spontaneous bacterial peritonitis. Intrahepatic immune cells play an important role in immune surveillance. The expansion of this phenotype which we observed in the liver could play a role in susceptibility to infection-mediated decompensation in liver disease. However, implying immune biology in liver diseases based on peripheral evaluation of immune markers, as opposed to full evaluation of liver infiltrating lymphocytes for example, has a potential for findings that are not always reflective of hepatic immunobiology. Utilizing single-cell molecular approaches have opened up new opportunities to surpass descriptive studies in health and disease. Ramachandran et al. have highlighted the utility of single-cell RNA-sequencing in dissecting mechanisms regulating human liver cirrhosis by uncovering 3 novel subsets involved in the pathogenesis of liver fibrosis including TREM2^+^CD9^+^ macrophages, ACKR1^+^ and PLVAP^+^ endothelial cells and PDGFRα^+^ collagen producing myofibroblasts [27]. Such studies deepen our understanding of pathogenic mechanisms of human disorders and provide novel insights into the design of therapeutic targets.

Altogether, we demonstrate for the first time a dysfunctional CD8^+^ *T* cell population in cirrhosis mainly defined by a predominant HLA-DR^+^CD8^+^ *T* cells subset displaying exhausted, activated and immune-modulatory properties. Specific mediators driving these alterations and the relevance of this population in clinical practice remain to be explored in further studies.

## Declaration of Competing Interest

The authors declare no competing interests.

## Appendix Supplementary materials

Image, application 1
