# Supplementary material for: CD8+T cells from patients with cirrhosis display a phenotype that may contribute to cirrhosis-associated immune dysfunction
Source: eBioMedicine. 2019 Oct 31;49:258–68. doi: 10.1016/j.ebiom.2019.10.011 (PMC6945243; doi:10.1016/j.ebiom.2019.10.011)
Supplement: Supplementary file 1 [file mmc1.docx]

**SUPPorting Methods**

**Exclusion criteria for recruited patients**

Exclusion criteria were the following: patients younger than 18 years; current or previous viral infection (Hepatitis A, B, C and E virus or Human Immunodeficiency Virus); malignancy; immune suppressive therapy except corticosteroids treatment for AAH (n=2). For matched circulating and peritoneal CD8^+^ T cells phenotyping, patients with concomitant Spontaneous Bacterial Peritonitis were excluded.

**Isolation of mononuclear and polymophonuclear cells**

Peripheral Blood Mononuclear Cells (PBMCs) were isolated from fresh blood by a density gradient centrifugation method using Ficoll^®^ Paque PLUS (GE Healthcare, Chicago, IL, USA). Ascites Mononuclear Cells (AMNCs) were isolated from patients after 10 minutes centrifugation of the ascitic fluid at 1500 rpm followed by a red cells lysis step with ACK lysis buffer (Thermo Fisher Scientific, Whattam, MA, USA). Fresh polymorphonuclear neutrophils (PMNs) were isolated from HV by a low density gradient centrifugation method using Polymorphprep™ (Tebu-bio, Le Perray en Yvelines, France) followed directly by magnetic-bead purification using CD66abce positive selection (Miltenyi Biotec, Bergisch Gladbach, Germany). Monocytes were isolated from PBMCs using CD14^+^ microbeads (Miltenyi Biotec, Bergisch Gladbach, Germany) according to manufacturer’s instructions.

**Phenotyping using flow cytometry**

CD8^+^ T cells from peripheral blood and from ascitic fluid were cell-surface phenotyped and intracellularly characterized using a twelve-color flow cytometry analysis, performed on LSRFortessa™ flow cytometer. Data were acquired using BD FACSDiva™ software (Becton Dickinson Ltd, Oxford, UK). Data analyses were performed using FlowLogic software (Inivai Technologies, Pty Ltd, Victoria, Australia).

Following cell Viability Dye staining (Thermo Fisher Scientific, Waltham, MA, USA), PBMCs and AMNCs were surface stained for the following T cell markers: CD3, CD8, CD45RO, CD45RA, CD62L, CCR7, HLA-DR, CD27, CD28, CD69, CD56, CTLA-4, PD-1, TIM-3, FAS and FASL. Baseline intracellular level of perforin and granzyme B were measured by intracellular staining performed on fixed and permeabilized cells according to the manufacturer’s instructions using the eBioscience™ FoxP3/Transcription Factor Staining Buffer Set (Thermo Fisher Scientific, Waltham, MA, USA). Regulatory T cells markers were characterized by the extracellular expression of CD25 and intracellular detection of FOXP-3. Natural Killer T (NKT) cells and Mucosal-Associated Invariant T (MAIT) cells were evaluated by additional surface staining of CD161 and either Vα24Jα18 or Vα7.2-Jα33 markers, respectively. Fluorescence minus one (FMO) were used as controls (Supplementary Figure 1A). Annexin V and 7-AAD apoptosis detection kit was used to characterize apoptosis in CD8^+^ T cells in whole blood from HV and cirrhotic patients according to the manufacturer’s protocol (BioLegend, San Diego, CA, USA).

**Immunohistochemistry**

Human liver tissues were obtained from liver explants of patients with cirrhosis undergoing orthotopic liver transplantation (n=8) or from colorectal cancer metastasis surgery serving as pathological controls (n=4). Formalin-fixed and paraffin-embedded tissues were stained manually as previously described^17^ using CD8 antibody (DAKO, dilution 1:50) and HLA-DR antibody (DAKO, dilution 1:100). Briefly, heat-induced epitope retrieval using EDTA Tris buffer pH 9, was followed by a cooling phase. Staining was then performed using the EnVision™ G|2 doublestain system *–* rabbit / mouse (DAB +/ permanent red) (Dako, Agilent Technologies, Santa Clara, CA, USA) according to the manufacturer’s instructions. The slides were processed as previously described to enable analysis by Nuance 3.0.2 multispectral imaging (PerkinElmer, Beaconsfield, UK) technology.[13] Cells were counted from 10 random high-power fields (HPF).

**CD8^+^ T cells isolation and culture**

CD8^+^ T cells were isolated from PBMCs using magnetic-based CD8^+^ T cell isolation kit (Miltenyi Biotec, Bergisch Gladbach, Germany). HLA-DR positive and their negative counterparts were obtained using the following multi-step isolation process. Firstly, we removed the CD16^+^ fraction using CD16^+^ cells magnetic selection (Miltenyi Biotec, Bergisch Gladbach, Germany). This was followed by a CD8^+^ T cells selection on the CD16^–^ fraction using CD8^+^ Multisort kit (Miltenyi Biotec, Bergisch Gladbach, Germany). Magnetic beads were then removed according to manufacturer’s protocol. Finally, HLA-DR^+^CD8^+^ T cells were selected using HLA-DR magnetic beads (Miltenyi Biotec, Bergisch Gladbach, Germany).

**CD8^+^ T cells conditioning in plasma**

Magnetically isolated CD8^+^ T cells were cultured at 5x10^5^ cells/well in RPMI 1640 (Thermo Fisher Scientific, Hemel Hempstead, UK) supplemented with 5% human AB serum (PAA laboratories Ltd, UK) at 37°C in 5% CO_2_. Conditioning of total CD8^+^ T cells in 25% of plasma from patients or HV was carried out for 3 days in the presence of IL-2 (rIL-2; 20 IU/mL) (Eurocetus Amsterdam, Netherlands).
